# Bone marrow failure may be caused by chromosome anomalies exerting effects on *RUNX1T1* gene

**DOI:** 10.1186/s13039-017-0352-2

**Published:** 2018-01-11

**Authors:** R. Valli, L. Vinti, A. Frattini, M. Fabbri, G. Montalbano, C. Olivieri, A. Minelli, F. Locatelli, F. Pasquali, E. Maserati

**Affiliations:** 1 Genetica Umana e Medica, Dipartimento di Medicina e Chirurgia, Università dell’Insubria, Varese, Italy; 2 Dipartimento di Onco-Ematologia Pediatrica, Ospedale Pediatrico Bambino Gesù, Roma, Università di Pavia, Pavia, Italy; 3 Istituto di Ricerca Genetica e Biomedica, CNR, Milan, Italy; 4 Unit of Haematopathology, European Institute of Oncology, Milan, Italy; 5 Genetica Medica, Fondazione IRCCS Policlinico S. Matteo and Università di Pavia, Pavia, Italy

**Keywords:** Severe aplastic anaemia, Pancytopenia, Chromosome structural anomalies, Chromosome 8, Chromosome 2, *RUNX1T1* gene

## Abstract

**Background:**

The majority of the cases of bone marrow failure syndromes/aplastic anaemias (BMFS/AA) are non-hereditary and considered idiopathic (80–85%). The peripheral blood picture is variable, with anaemia, neutropenia and/or thrombocytopenia, and the patients with idiopathic BMFS/AA may have a risk of transformation into a myelodysplastic syndrome (MDS) and/or an acute myeloid leukaemia (AML), as ascertained for all inherited BMFS. We already reported four patients with different forms of BMFS/AA with chromosome anomalies as primary etiologic event: the chromosome changes exerted an effect on specific genes, namely *RUNX1*, *MPL*, and *FLI1*, leading to the disease.

**Results:**

We report two further patients with non-hereditary BM failure, with diagnosis of severe aplastic anaemia and pancytopenia caused by two different constitutional structural anomalies involving chromosome 8, and possibly leading to the disorder due to effects on the *RUNX1T1* gene, which was hypo-expressed and hyper-expressed, respectively, in the two patients. The chromosome change was unbalanced in one patient, and balanced in the other one.

**Conclusions:**

We analyzed the sequence of events in the pathogenesis of the disease in the two patients, including a number of non-haematological signs present in the one with the unbalanced anomaly. We demonstrated that in these two patients the primary event causing BMFS/AA was the constitutional chromosome anomaly. If we take into account the cohort of 219 patients with a similar diagnosis in whom we made cytogenetic studies in the years 2003–2017, we conclude that cytogenetic investigations were instrumental to reach a diagnosis in 52 of them. We postulate that a chromosome change is the primary cause of BMFS/AA in a not negligible proportion of cases, as it was ascertained in 6 of these patients.

**Electronic supplementary material:**

The online version of this article (10.1186/s13039-017-0352-2) contains supplementary material, which is available to authorized users.

## Background

Bone marrow failure syndromes/aplastic anaemias (BMFS/AA) are a heterogeneous group of disorders characterized by the inability of the bone marrow (BM) to produce an adequate number of blood cells. The consequence is peripheral blood (PB) cytopenia, which may be uni-, bi-, or trilinear, resulting in anaemia, neutropenia and/or thrombocytopenia. The BMFS/AA are inherited with a Mendelian pattern in about 15–20% of the patients: in these inherited BMFS (IBMFS) a number of extra-haematological signs are present, and many causative gene mutations have been identified [1]. The majority of the non-hereditary cases are considered idiopathic because their etiology is not known [2]. A risk of transformation into myelodysplastic syndrome (MDS) and/or acute myeloid leukaemia (AML) is ascertained for all IBMFS [3], and it may affect also patients with idiopathic BMFS/AA. This risk is well established for long-term survivors of acquired idiopathic AA [4], and it may be present also in different conditions belonging to the group defined above of BMFS/AA, which share almost all the haematological and clinical characteristics of IBMFS except the monogenic etiology.

We have already reported four patients with different forms of BMFS/AA with chromosome anomalies as primary etiologic event. They were two patients with complex structural rearrangements of chromosome 21, constitutional in one of them and acquired in BM in the other one, causing the disruption or the loss of the gene *RUNX1*, which was therefore hypo-expressed and lead to a Severe AA (SAA) in one patient, and to a congenital thrombocytopenia in the other one [5]. Another patient showed a paracentric inversion of a chromosome 1 as acquired clonal anomaly in the BM: we postulated that it caused AA due to a position effect acting on the gene *MPL*, severely hypo-expressed, with a final diagnosis of Congenital Amegakaryocytic Thrombocytopenia (CAMT) [5]. The clonal anomaly in the BM of one further patient was a complex unbalanced translocation with partial monosomy of the long arm of chromosome 11 implying the loss of the *FLI1* gene*,* consequently hypo-expressed and leading to diagnosis of Paris-Trousseau type thrombocytopenia [6].

We report here two further patients with non-hereditary BM failure, with diagnosis of SAA and pancytopenia, respectively, caused by two different constitutional structural anomalies involving chromosome 8, and leading to the disorder due to effects on the *RUNX1T1* gene. We postulate that a chromosome change is the primary cause of BMFS/AA in a not negligible proportion of cases.

### Clinical reports

#### Patient 1

Female child, born in 2009 from non-consanguineous healthy parents; her birthweight was Kg 3.200. Two elder sibs were healthy. No relevant perinatal problems were present, but an ostium secundum atrial septal defect was diagnosed at 1 month of life: the right heart overload led then to surgical treatment, in January 2015.

She was admitted to hospital firstly at 8 months due to growth delay (weight, height, and cranium circumference < 3rd centile), psychomotor retardation, and facial dysmorphisms. In July 2012 she was hospitalized due to seizure episodes, and a severe non-haemolitic anaemia was noticed (Hb 4.7 g/dL). BM smear had a normal appearance, but the biopsy showed a hypoplastic marrow with slight dysplastic signs. BM cell cultures showed relevant reduction of all haemopoietic progenitors. The Diepoxybutane (DEB) test excluded Fanconi Anaemia (FA), and also Blackfan Diamond Anaemia was excluded. Her spleen was enlarged at echo-scan. Her radii were normal at Rx-scan, as normal were metabolic tests and a magnetic resonance tomography of her head. A diagnosis of AA was made, and therapy required monthly transfusions.

BM morphology was checked in November 2012 and in May 2013: it was hypocellular, with signs of trilinear dysplasia that worsened slightly in time, although the erythroid series showed some signs of recovery. In May 2014 the BM picture was substantially unchanged, with a hypocellular marrow and some dysplastic signs that did not reach the criteria to change the diagnosis to Refractory Cytopenia. Blood test in April 2016 showed: Hb 10.6 g/dL, WBC 3.9 × 10^9^/L, platelets 173 × 10^9^/L.

In November 2013 an echo-scan revealed a left kidney reduced (< 3rd centile) and a duplex kidney at the right (mass > 97th centile).

Epileptic seizures were observed three times from 2012 to 2014. Some epileptic anomalies were present at EEG, the last in October 2015, but no episodes took place after 2014. A neuropsychological examination in 2014 showed a border-line cognitive level, with normal speech, but under logopaedic treatment.

#### Patient 2

Female child, born in 2013 by Caesarian section from non-consanguineous healthy parents; her birthweight was Kg 2.900. Prenatal diagnosis performed through amniocentesis had shown the presence of a constitutional chromosome anomaly, interpreted as a balanced translocation involving the short arm of chromosome 2 and the long arm of chromosome 8. She has a healthy elder sister.

At 7 months of age she was admitted to hospital due to fever, and pancytopenia was diagnosed: the blood count showed Hb 5 g/dL, WBC 4.8 × 10^9^/L with 0.180 × 10^9^/L neutrophils, platelets 74 × 10^9^/L. A panel of virological tests gave negative results. BM examination showed arrested maturation with dyserythropoiesis. In November 2013 her general conditions were good, her growth was normal both as to weight and height, repeated microbiological and virological tests were negative. BM cell cultures failed to show any abnormal results, whereas the trilinear cytopenia persisted. The DEB test excluded FA. Transfusions were given, and the administration of Ig led to increase the platelet number, which varied variously in the following months. In December 2013, her blood count showed Hb 9.8 g/dL, WBC 4.210 × 10^9^/L with 0.210 × 10^9^/L neutrophils, platelets 102 × 10^9^/L. Ig administration and RBC transfusions were periodically given in the following months, Hb and platelets increased while neutropenia persisted. A blood count in May 2014 showed Hb 10.9 g/dL, WBC 4.3 × 10^9^/L with 0.390 × 10^9^/L neutrophils, platelets 94 × 10^9^/L, and in March 2015 Hb 12.2 g/dL, WBC 5.7 × 10^9^/L with 1.830 × 10^9^/L neutrophils, platelets 176 × 10^9^/L.

## Results

### Patient 1

**Fig. 1:**
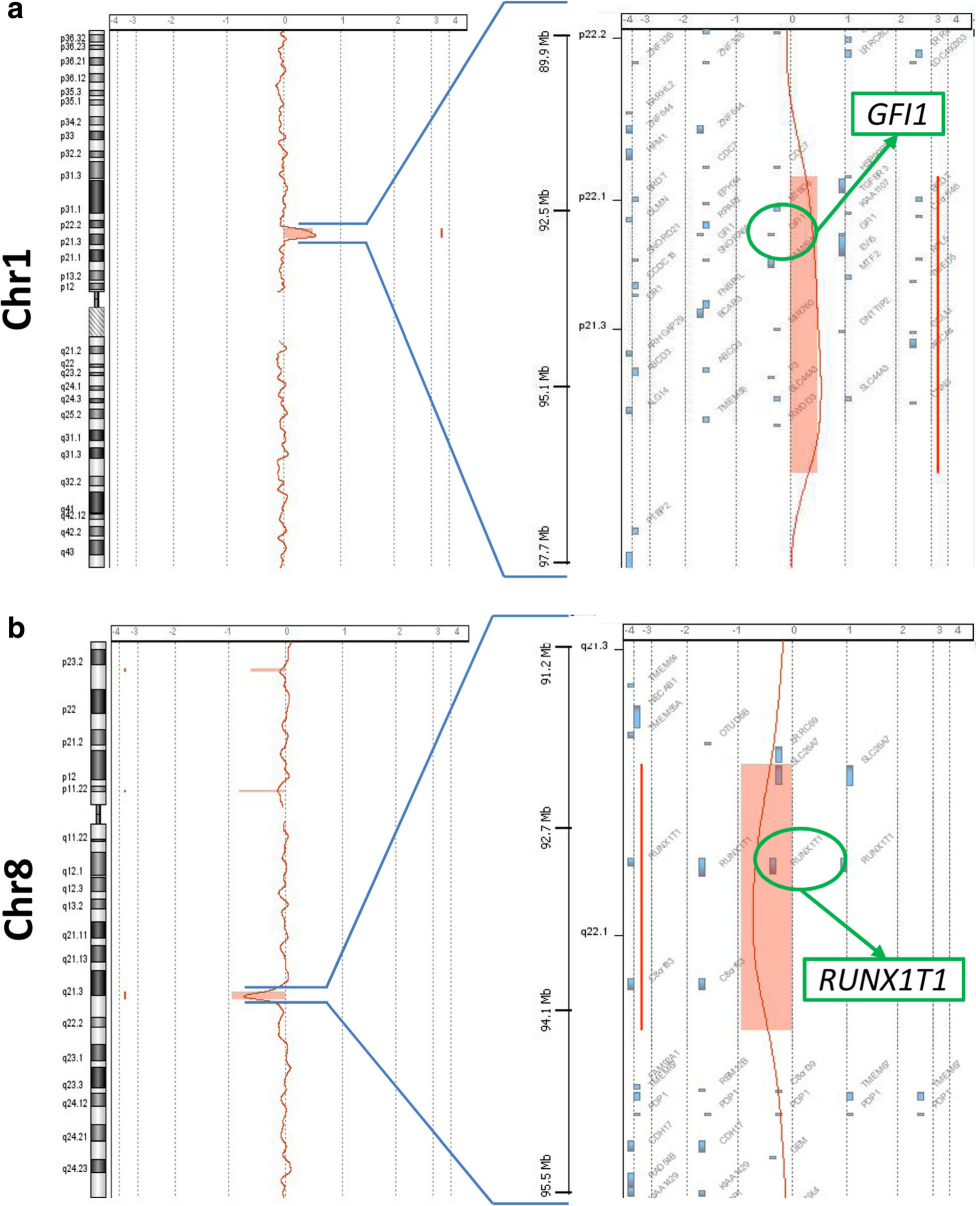
Patient 1: a-CGH profiles of chromosomes 1 (**a**) and 8 (**b**). In the enlarged view (at the right) the locations of the genes *GFI1* (chromosome 1) and *RUNX1T1* (chromosome 8) in evidence (arrows)

**Fig. 2:**
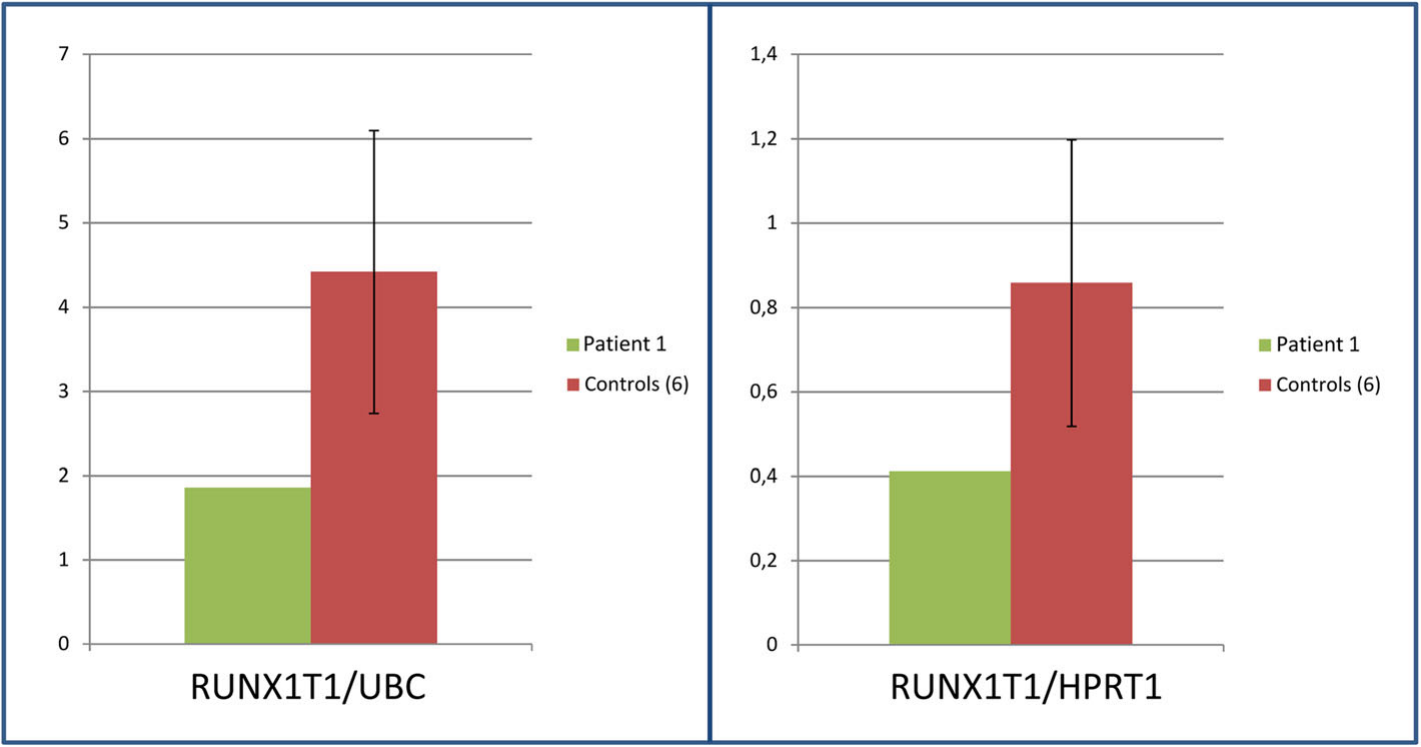
Relative expression of *RUNX1T1* in the BM of patient 1. The green bars refer to the patient and the red bars to 6 controls’ average values: two control housekeeping genes were used, *UBC* (left) and *HPRT1* (right). Standard error is shown for controls

Chromosome analysis performed with QFQ-banding technique on PB stimulated cultures (in 2012 and 2014), on BM (in 2013 and 2014), and on the lymphoblastoid cell line consistently showed a normal karyotype. The a-CGH performed on DNA from PB revealed two imbalances: a duplication of the short arms of chromosome 1 of 4.304 Mb, from 92,091,957 to 96,396,550 bp (genome assembly hg19) (Fig. 1a), and a deletion of the long arms of chromosome 8 of 2.045 Mb, from 92,249,936 to 94,294,548 bp (Fig. 1b). Fluorescent In Situ Hybridization (FISH) with a commercial probe designed to detect the translocation t(8;21) (Table 1) showed that the signal of the *RUNX1T1* gene (alias *ETO*) was absent from the deleted chromosome 8 in mitoses from PB. On the same material the painting with a whole chromosome 1 library covered entirely the duplicated chromosome 1, without any signal elsewhere. The expression of *RUNX1T1*, evaluated by real-time on BM drawn in 2014, was significantly lower than controls (Fig. 2).

**Table 1:** Probes and libraries used for FISH

Pt	Chromosome	Probes/libraries	Band localization	Genomic Localization (bp) (hg19)
1	8	AML1/ETO^a^ WCP 1^b^	designed for t(8;21) Whole chromosome paint library	–
2	2	WCP 2^b^ CTD-2314L14^c^ CTD-2599D22^c^ ALK^d^ CTD-3028D11 ^c^ CTD-2232H14^c^ CTD-2566H13^c^ CTD-2532N8^c^ CTD-2371H10^c^ CTD-2382F21^c^ CTD-2052G24^c^ CTD-3058K15^c^	Whole chromosome paint library 2p23.3 2p23.2 2p23.2–23.1 2p23.1 2p22.2 2p22.2–22.1 2p21.3 2p21.1 2p16.3 2p15 2p15–14	27,436,476–27,577,216 bp 29,678,603–29,819,833 bp - 31,532,615–31,667,611 bp 36,717,490–36,828,693 bp 38,563,970–38,725,504 bp 42,157,290–42,301,116 bp 46,582,036–46,715,607 bp 48,326,711–48,434,143 bp 61,463,234–61,600,608 bp 64,034,477–64,232,972 bp
	8	WCP 8^b^ AML1/ETO^a^ CTD-2341G19^c^ CTD-2246G22^c^ CTD-3245G9^c^ CTD-2330N9^c^	Whole chromosome paint library designed for t(8;21) 8q21.13 8q21.3 8q21.3 8q21.3	- 83,886,651–83,982,215 bp 87,885,140–87,980,460 bp 91,740,722–91,959,017 bp 92,778,106–92,922,619 bp

^a^AML1/ETO translocation, dual fusion probe, Cytocell Technologies, Cambridge, UK

^b^WCP, Cytocell Technologies, Cambridge, UK

^c^BAC probes, Thermo Fisher Scientific, Waltham, MA, USA

^d^ZytoLight SPEC ALK Dual Color Break Apart probe, Zytovision GmbH, Bremerhaven, Germany

The karyotype of the parents was normal, and the result of a-CGH, performed on mother’s vs. father’s DNA, showed no significant deviation.

### Patient 2

**Fig. 3:**
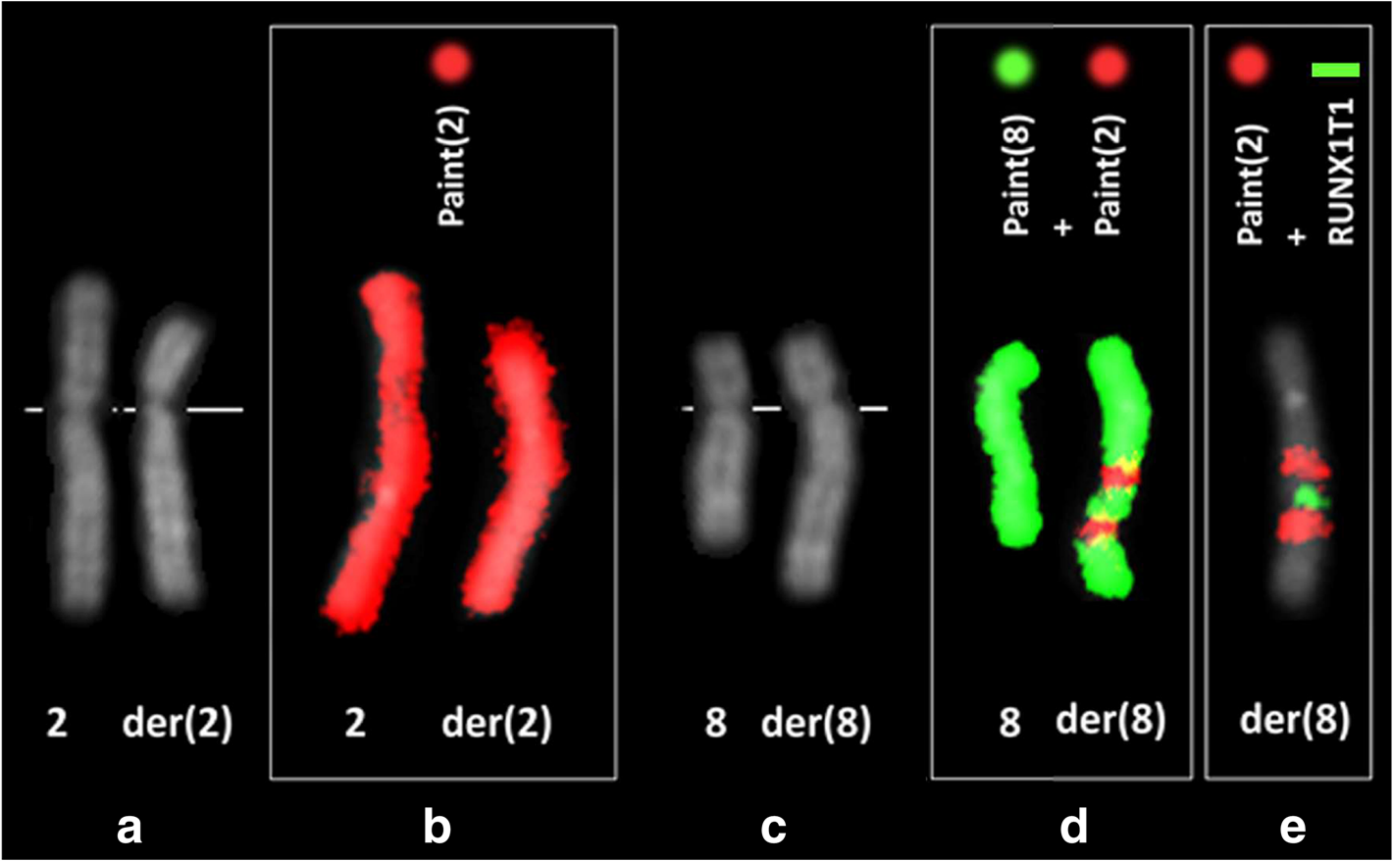
Cut-out of the chromosomes involved in the rearrangement in patient 2. In **a** and **c** the Q-banded chromosomes (normal 2 and 8 at the left). In **b** the painting result on the normal chromosome 2 (left) and on the rearranged one (right) with the chromosome 2 library. In **d** the result of dual color painting with chromosomes 2 and 8 libraries on the normal chromosome 8 (left) and on the rearranged one (right). In **e** the dual color FISH with the chromosome 2 library (red) and a probe recognizing the entire sequence of the *RUNX1T1* gene, part of the system to detect the AML1/ETO translocation (Table 1) (green)

Chromosome analysis performed with QFQ-banding technique on PB stimulated cultures (in 2013 and 2014), on BM (in 2013), and on the lymphoblastoid cell line consistently showed a complex anomaly, already found at prenatal diagnosis performed elsewhere on amniotic fluid, and interpreted as a translocation t(2;8). Painting by FISH with libraries of chromosomes 2 and 8 (Table 1) showed that the anomaly consisted in fact of two separate insertions of material from the short arms of chromosome 2 into two points of the long arms of chromosome 8 (Fig. 3). FISH with a probe recognizing the entire sequence of the *RUNX1T1* gene (Table 1) showed that it was intact and included in the segment of chromosome 8 between the two insertions (Fig. 3e). The a-CGH performed on DNA from BM showed normal results, confirming that the rearrangement did not lead to any imbalances.

We then performed several dual color FISH with the probes of chromosomes 2 and 8 listed in Table 1 in various combinations to define precisely the breakpoints. The results, compared with the morphological appearance of the rearranged chromosomes, permitted to indicate the linear composition of the derivatives der(2) and der(8) as follows: 2pter→2p23.3::2p16.3→2qter; 8pter→8q21.12::2p16.3→2p22.2::8q21.12→8q22.2::2p23.3→2p22.2::8q22.2→8qter (Figures in Additional files 1 and 2).

**Fig. 4:**
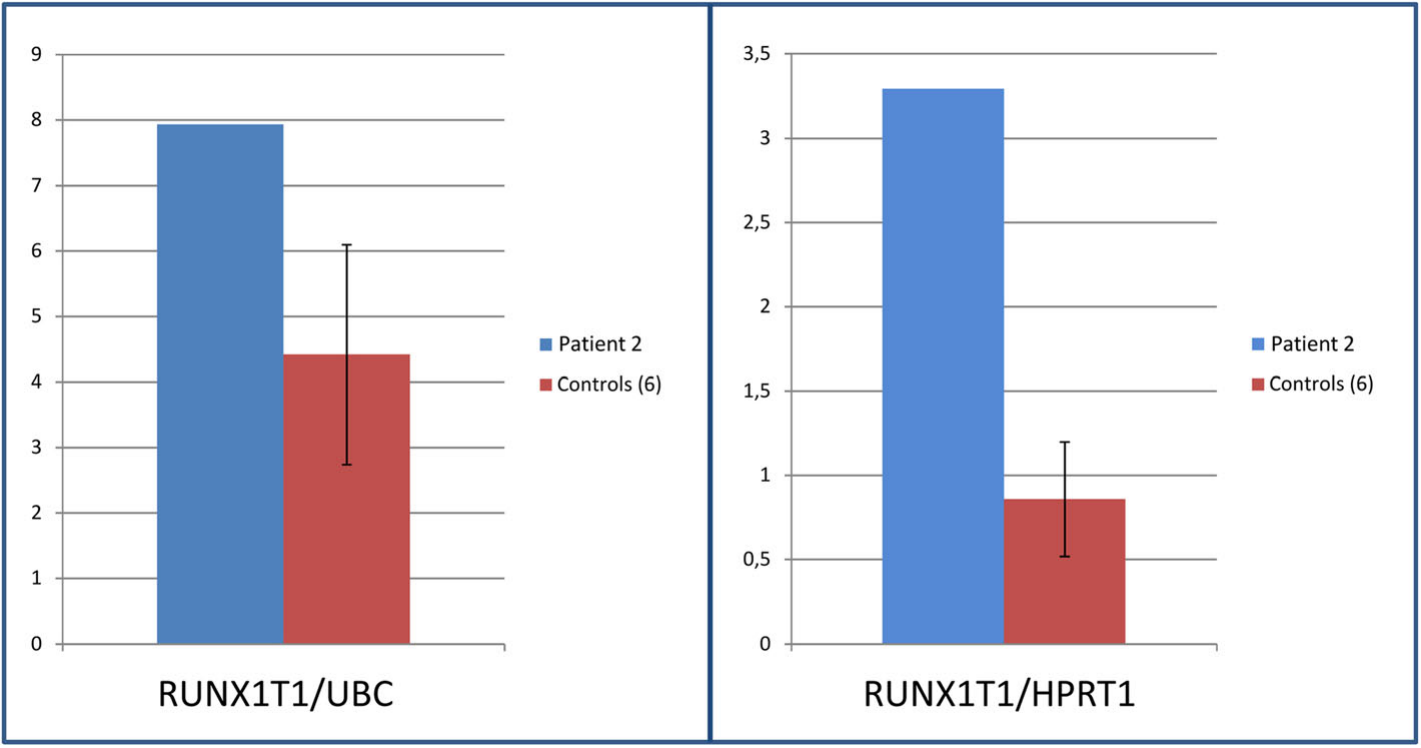
Relative expression of *RUNX1T1* in the BM of patient 2. The blue bars refer to the patient and the red bars to 6 controls’ average values: two control housekeeping genes were used, *UBC* (left) and *HPRT1* (right). Standard error is shown for controls

The expression of *RUNX1T1*, evaluated by real-time on BM drawn in 2014, was significantly higher than controls (Fig. 4).

The karyotype of the parents and of the sister was normal.

## Discussion

About 80–85% of BMFS/AA are considered idiopathic as the primary cause remains unknown [2]. We have already reported four patients with an initial diagnosis of idiopathic BMFS/AA, who were shown to bear a chromosome anomaly, either as constitutional change, or clonal in the BM, which led to the disorder through effects on genes localized in the chromosomes involved and their deregulated expression [5, 6]. The final diagnosis became SAA and congenital neutropenia in two patients, in whom the expression of the *RUNX1* gene (and possibly other uninvestigated genes) was reduced: both these conditions are usually not hereditary. In one case the final diagnosis became CAMT, likely due to effects on the *MPL* gene: this disorder is inherited usually as an autosomal recessive trait. Thrombocytopenia of the Paris-Trousseau type (TCPT) was the final diagnosis of another patient, due to the loss of the *FLI1* gene caused by a complex unbalanced translocation: this condition is usually not transmitted as a monogenic trait, but it is due to subtle deletions of the region of chromosome 11 containing the *FLI1* gene. The chromosome change is inherited from a parent in very few reported cases of TCPT [7].

We report here two further patients with a similar pathogenetic pathway, in whom we postulate that a chromosome anomaly was the primary event with subsequent deregulated expression of the *RUNX1T1* gene.

The strategy of analysis that we followed gave the proof that *RUNX1T1* expression dysregulation was the cause of the bone marrow failure in these two patients. We made a list of all genes included in the regions involved in the imbalances of our patient 1, and of genes in regions with proximity to the breakpoints of patient 2 (genome assembly hg19) [8]. These lists included 45 genes in the region duplicated of chromosome 1, and 6 genes in the region of chromosome 8 deleted in patient 1. As to patient 2, the lists included 227 genes in the two inserted regions of chromosome 2, and 139 genes in the region of chromosome 8 left between the two insertions and in the adjacent regions above and below the insertions (bands 8q21.12 – 8q22.2). We selected from these lists the genes known to be relevant in haematopoiesis. Thus, we arrived to the genes *GFI1*, on chromosome 1, and *RUNX1T1*, on chromosome 8, and we analyzed their expression. Data on the function of *RUNX1T1* are scarce in literature: it encodes a member of the myeloid translocation gene family, which interacts with DNA-bound transcription factors and recruit a range of corepressors to facilitate transcriptional repression, playing an important role in haematopoiesis, myogenesis [9], and neuronal differentiation [10]. Most reports on *RUNX1T1* are related to the translocation t(8;21)(q22;q22), which is one of the most frequent acquired chromosome changes in the BM of patients with AML. This translocation gives a chimeric gene made up of the 5′-region of the runt-related transcription factor 1 gene (*RUNX1*) fused to the 3′-region of *RUNX1T1*. The chimeric protein so produced interferes with the expression of a number of genes relevant to normal haematopoiesis [11].

The sequence of pathological events that we postulate for our patient 1 is as follows: constitutional unbalanced chromosome anomaly involving the chromosomes 1 and 8, not detectable at standard chromosome analysis, but precisely identified by a-CGH. This anomaly led to the duplication of a segment of 4.304 Mb in the bands p22.1–p21.3 of the short arm of chromosome 1 (Fig. 1a), and to the deletion of a 2.044 Mb segment in the band q22.1 of the long arm of chromosome 8 (Fig. 1b). The gene *RUNX1T1* is in this region of chromosome 8 (Fig. 1b): its haploinsufficiency led to hypo-expression in BM (Fig. 2), which, in turn, caused the SAA. In the duplicated region of chromosome 1, the only gene known to play a role in haematopoiesis is *GFI1* (Fig. 1a), which functions as a transcription repressor [12]. It would be speculative to link the duplication of *GFI1* with the SAA of our patient, but in any case we analysed its expression and found it normal in comparison with six controls (Figure in Additional file 3). On the contrary, the hypoexpression of *RUNX1T1* is conceivable to deregulate the expression of other genes leading to SAA.

Extra-haematological symptoms of patient 1 include developmental and psychomotor delay, facial dysmorphisms, slight intellectual disability, rare seizures episodes, ostium secundum atrial septal defect, and kidney malformations. They are due to the chromosome imbalances of chromosomes 1 and 8, but a reliable comparison with patients with similar cytogenetic anomalies is not feasible, although some signs of our patient are common to similar reported cases. If we look at literature based on standard cytogenetics, we may compare our patient with cases as those reviewed by Utkus et al. [13] with duplications of at least part of band 1p21 (but with no imbalance of chromosome 8). If we take into account cases defined at DNA base-pair level, the DECIPHER web-based database of Chromosome imbalances [14] includes 19 patients with duplications of chromosome 1 at least partially overlapping with the duplication of our patient, and 13 patients with deletions of chromosome 8 at least partially overlapping with the deletion. Some clinical signs of our patient are present in some of these cases, although the clinical definition of the reported patients is often somehow generic: intellectual disability, often moderate (10/32 patients), developmental delay (2/32), congenital heart defects (4/32) (including one case of interatrial, but also interventricular, defect in one patient with deletion of 8q), seizures (2/32), dysmorphisms (6/32). However, the duplication and the deletion of these patients are not identical to the imbalances of our patient, and none had both the imbalances of chromosomes 1 and 8. A number of patients with constitutional deletion of the long arm of chromosome 8 have been reported, with loss of material which included also the *RUNX1T1* gene. In these reports, however, as the ones of Zhang et al. and Allanson et al. [10, 15], the focus is almost exclusively on dysmorphisms/malformations, intellectual disability, and growth problems, no laboratory data at all are given and possible haematological issues may have been overlooked.

The sequence of pathological events that we postulate for our patient 2 is as follows: constitutional complex and balanced chromosome rearrangement involving chromosomes 2 and 8, with two contiguous but separate segments of the short arm of chromosome 2 (p23.3-p22.2, p22.2-p16.3) inserted in two bands of the long arms of chromosome 8 (q21.12, q22.2) (Fig. 3 and in Additional files 1 and 2). No loss or gain of chromosome material was confirmed by a-CGH. The *RUNX1T1* gene was shown to be intact, and it is normally located between the two insertions. It was highly hyper-expressed in the BM (Fig. 4): we believe that this led to BM failure and pancytopenia. Also the hyperexpression of *RUNX1T1* is apt to deregulate the expression of other genes leading to SAA.

We performed also whole transcriptome analysis on BM of both patients, and we found no other genes significantly over- or hypoexpressed (data not shown).

A pathogenetic pathway similar to that of our patients led to Diamond-Blackfan anaemia (DBA) in a reported boy with a de novo constitutional microdeletion of the band q13.2 of chromosome 19, where the gene *RPS19* is located [16]. This gene is known to cause DBA, and also in this patient the primary event leading to the BMFS was the chromosome anomaly, which caused also non-haematological features.

## Conclusions

In the period 2003–2017 we performed cytogenetic analyses in a heterogeneous cohort of 219 pediatric patients with BMF/AA during the evaluations made to reach a diagnosis. We found chromosome lesions in the BM or in the PB of 55 of these patients. The majority of them, 37, were diagnosed as affected by Fanconi Anaemia, as they showed chromosome breaks in PB cultures, in particular with the DEB test. In 9 patients with monosomy 7 or trisomy 8 in BM the final diagnosis was MDS [17, 18]. One patient with acquired trisomy 8 was then diagnosed as affected by Congenital Amegakaryocytic Thrombocytopenia (CAMT, OMIM # 604998) caused by biallelic mutations of the *MPL* gene [19]. One patient with an isochromosome of the long arm of a chromosome 7 was then diagnosed as affected by Shwachman-Diamond syndrome, as he was found to be compound heterozygote for mutations of the *SBDS* gene [20]. One patient with a translocation t(8;17)(p21;q25) acquired in BM, was a case of Diamond-Blackfan anaemia (DBA). Then there are the four patients mentioned above, in whom the primary event leading to BMF/AA was a chromosome constitutional or acquired anomaly, in absence of morphological evidence of frank MDS, acting through effects on the *RUNX1*, *MPL*, or *FLI1* genes and leading to the different final diagnoses already mentioned [5, 6].

With the two patients reported here, the total number of cases with BMF/AA bearing a chromosome lesion is 55 out of 219, and the chromosome anomaly, either constitutional or acquired, was the primary etiologic event in 6 of them. We might add two further patients of our cohort in whom the pattern of etiology and pathogenesis could be again similar, although we were not able to reach a firm conclusion in this sense due to the lack of informative material to analyse. They are the case of DBA mentioned above, with a clonal translocation in BM involving chromosome 8 short arm, where a causative gene not yet identified is localized [21], and a 10-year-old patient with AA who had a normal karyotype when we had the opportunity to study her, but in whom a previous analysis, as far as we were informed, showed an acquired deletion of the long arm of chromosome 8 in the BM, approximately in the region of the *RUNX1T1* gene (personal communication from Dr. Marco Zecca, Pavia, Italy, and Drs. Svetlana Donska, Larysa Peresada and Elena Kreminska, Kyiv, Ukraine).

The considerations above show that cytogenetic analyses may often be instrumental to reach a correct diagnosis in BMFS/AA, and that a chromosome change, both numerical or structural, constitutional or clonal, is the primary cause of BMFS/AA in a small but certainly not negligible proportion of cases.

## Methods

Chromosome analyses were repeatedly performed in the two patients with routine methods and QFQ-banding technique on BM direct preparations and 24-48^h^ cultures, on PB unstimulated and PHA-stimulated cultures, and on cells from lymphoblastoid cell lines established by Epstein-Barr virus (EBV) infection. Routine methods were also applied for chromosome analysis of the patients’ parents and of a sister of patient 2.

FISH was done on metaphases by standard procedures with different probes and libraries to define the chromosome anomalies, both in patients 1 and 2. All probes and libraries used for FISH assays are listed in Table 1.

The a-CGH was performed with the 244 K genome-wide system (Agilent Technologies Inc., Santa Clara, CA, USA), according to the manufacturer’s instruction on DNA from PB of patient 1 and her parents, on DNA from BM of patient 2 and her parents.

The DNA was extracted using the Qiagen Flexigene kit (QIAGEN GmbH, Hilden, Germany), and competitor DNA was purchased from Agilent as part of the labeling kit. Slides were scanned using Agilent’s microarray scanner G2565CA and microarray images were analysed using Agilent’s Feature Extraction 12.0.2.2 software, and by Agilent’s Genomic Workbench software (7.0.4.0). All map positions in the results refer to the genome assembly hg19.

The relative expression of the *RUNX1T1* gene was evaluated in both patients on RNA from total BM using Applied Biosystems ABI 7000 real-time thermocycler (Life Technologies Corporation, Carlsbad, California, USA), and the results were compared with RNA from BM of 6 age-matched healthy control subjects who donated haematopoietic cells for transplantation of a relative.

The assay was performed with Applied Biosystems Taqman system: we used #Hs00231702_m1 primers/probe-set for *RUNX1T1* transcript, and #Hs_00824723_m1, for Ubiquitin C (*UBC*), and #Hs02800695_m1, for Hypoxanthine Phosphoribosyltransferase 1 (*HPRT1*), primers/probe-sets as control house-keeping genes, as suggested for analysis on BM by Vandesompele et al. [22]. Relative expressions were calculated by the standard ΔΔCt method [23].

## Additional files

Additional file 1: Karyotype of patient 2. (JPEG 608 kb)

Additional file 2: Ideograms of chromosome 2, der(2), 8, and der(8) of Patient 2, as defined by chromosome analysis and a-CGH. In red the region of chromosome 2 inserted in the der(8) chromosome, in orange the region of chromosome 2 that remains on the der(2) chromosome above the breakage. (JPEG 964 kb)

Additional file 3: Relative expression of *GFI1* in the BM of patient 1. The green bars refer to the patient and the red bar to 6 controls’ average values: two control housekeeping genes were used, *UBC* (left) and *HPRT1* (right). Standard error is shown for controls. (JPEG 528 kb)

## Abbreviations

AA: Aplastic anaemias
a-CGH: Array comparative genomic hybridization
AML: Acute myeloid leukaemia
BM: Bone marrow
BMFS: Bone marrow failure syndromes
CAMT: Congenital amegakaryocytic thrombocytopenia
DBA: Diamond-blackfan anaemia
DEB: Diepoxybutane
EBV: Epstein-Barr virus
FA: Fanconi anaemia
FISH: Fluorescent in situ hybridization
FLI1: Friend leukemia virus integration 1
GFI1: Growth factor-independent 1
IBMFS: Inherited bone marrow failure syndromes
MDS: Myelodysplastic syndrome
MPL: Myeloproliferative leukemia virus oncogene
PB: Peripheral blood
QFQ: Q banding by fluorescence and quinacrine
RPS19: Ribosomal protein S19
RUNX1: Runt-related transcription factor 1
RUNX1T1: Runt-related transcription factor 1, translocated to, 1
SAA: Severe aplastic anaemia
TCPT: Thrombocytopenia Paris-Trousseau type
