# Supplementary figures and images for: Bone marrow failure may be caused by chromosome anomalies exerting effects on RUNX1T1 gene
Source: Mol Cytogenet. 2018 Jan 11;11:2. doi: 10.1186/s13039-017-0352-2 (PMC5765665; doi:10.1186/s13039-017-0352-2)

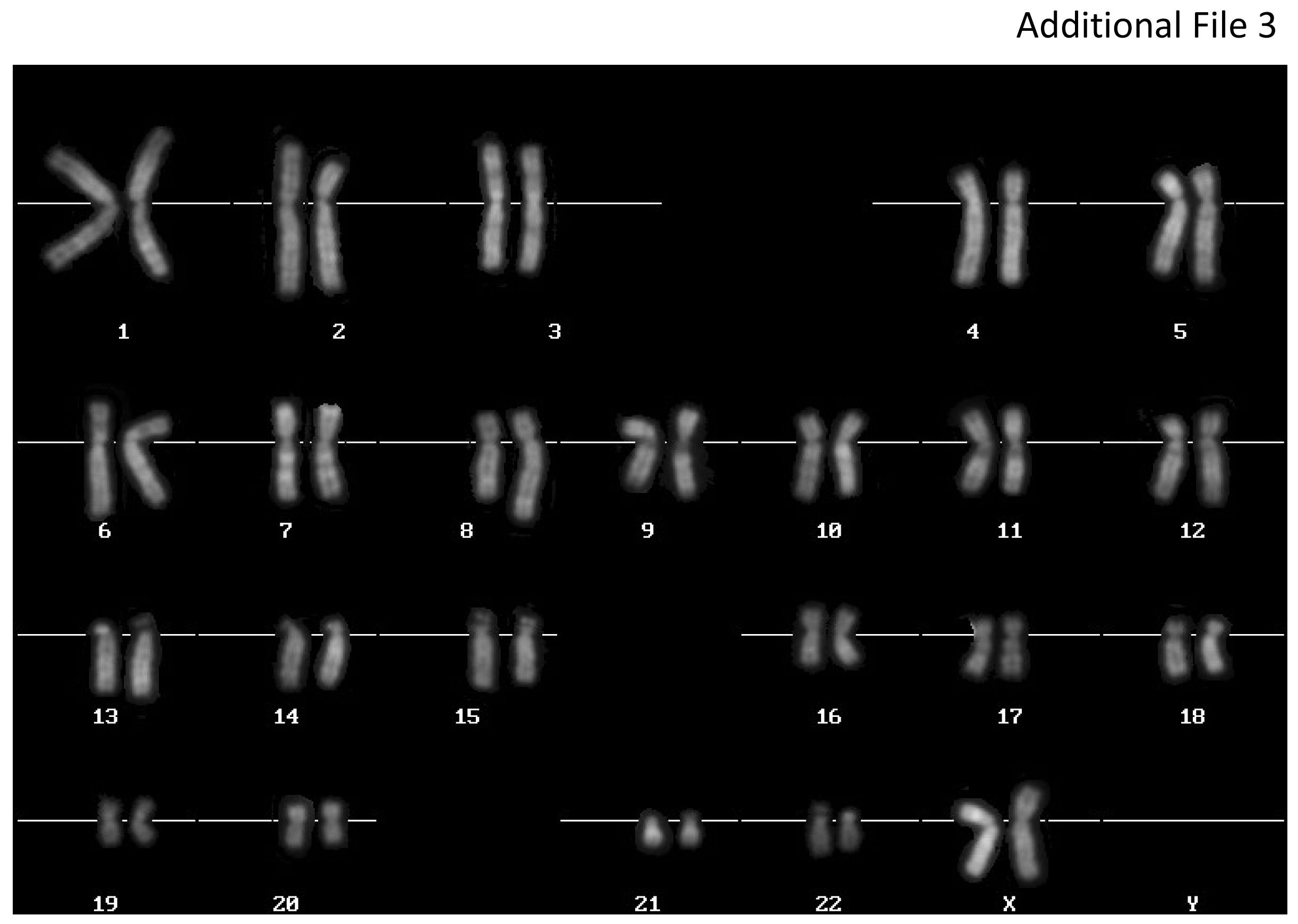

Supplement: Supplementary file 1 — Karyotype of patient 2. (JPEG 608 kb) [file 13039_2017_352_MOESM1_ESM.jpg]

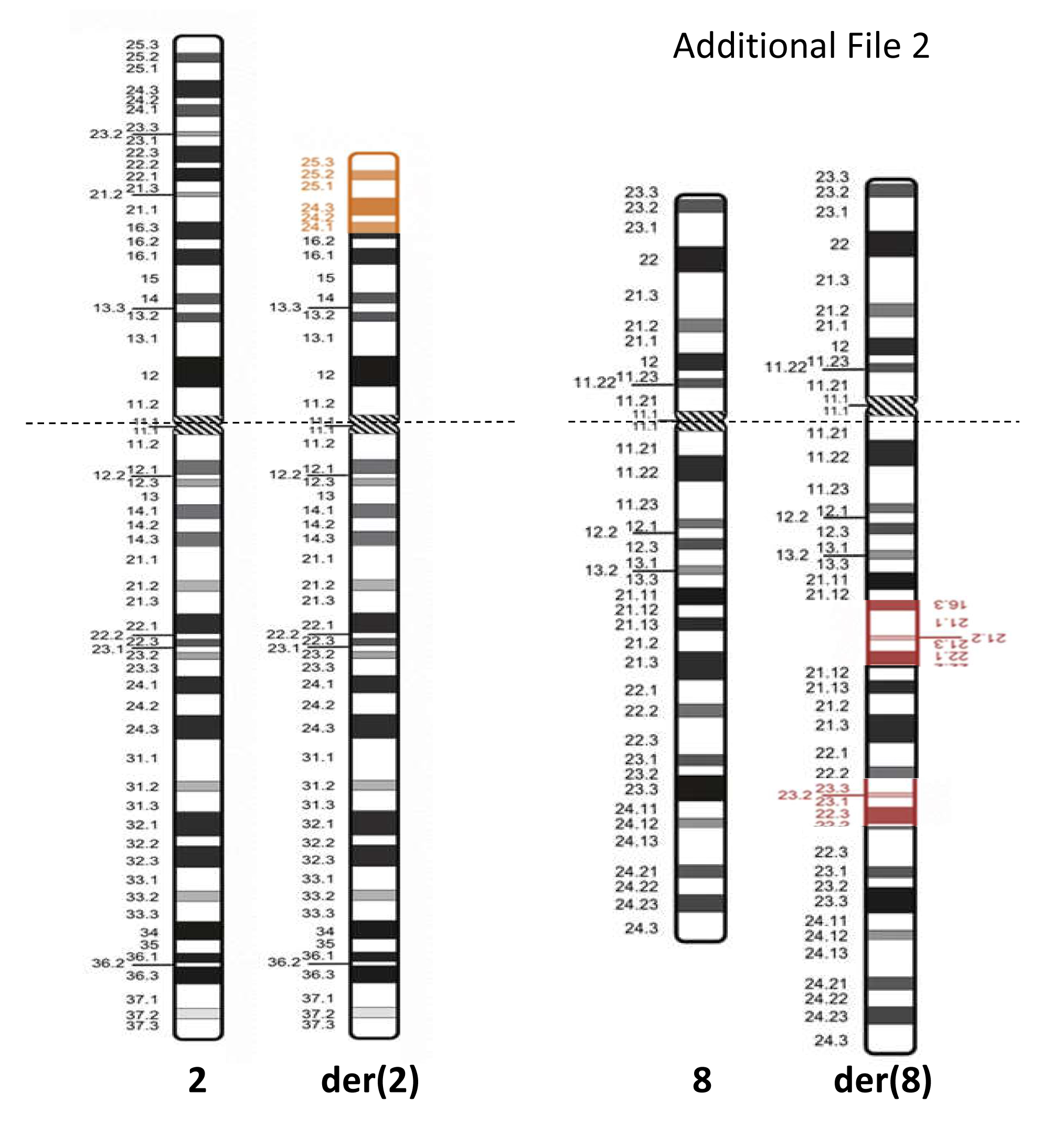

Supplement: Supplementary file 2 — Ideograms of chromosome 2, der(2), 8, and der(8) of Patient 2, as defined by chromosome analysis and a-CGH. In red the region of chromosome 2 inserted in the der(8) chromosome, in orange the region of chromosome 2 that remains on the der(2) chromosome above the breakage. (JPEG 964 kb) [file 13039_2017_352_MOESM2_ESM.jpg]

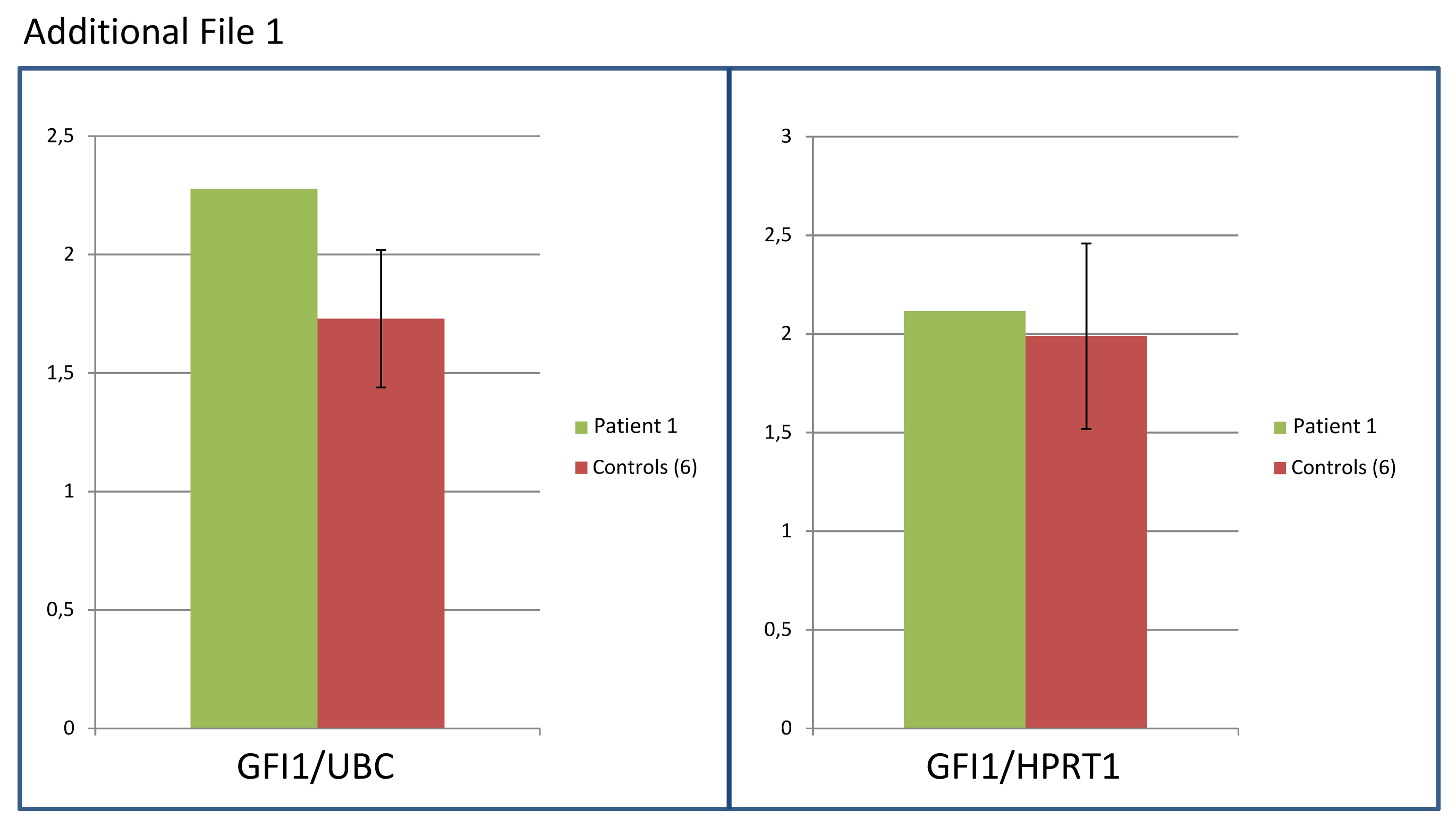

Supplement: Supplementary file 3 — Relative expression of GFI1 in the BM of patient 1. The green bars refer to the patient and the red bar to 6 controls’ average values: two control housekeeping genes were used, UBC (left) and HPRT1 (right). Standard error is shown for controls. (JPEG 528 kb) [file 13039_2017_352_MOESM3_ESM.jpg]
